# Simulation of the Parameters Effecting the Water Quality Evolution of Xuanwu Lake, China

**DOI:** 10.3390/ijerph18115757

**Published:** 2021-05-27

**Authors:** Min Pang, Weiwei Song, Yuan Liu, Yong Pang

**Affiliations:** 1 School of Environmental Science and Engineering, Southern University of Science and Technology, Shenzhen 518055, China; mia_pang@sina.com; 2 Key Laboratory of Integrated Regulation and Resource Development on Shallow Lakes, Ministry of Education, College of Environment, Hohai University, Nanjing 210098, China; 3 Jiangsu Environmental Engineering Technology Company Limited, Nanjing 210036, China; zyx950427@163.com

**Keywords:** water quality improvement, influence parameters, model simulation, Xuanwu Lake

## Abstract

After years of water environment improvement, China’s water quality has improved to some extent in recent years. However, different water areas have different characteristics of water pollution. The paper used mathematical models to investigate the influence of different parameters on the water quality of Xuanwu Lake, China. The predominant focus was on the nutrients concentration due to changing the amount of pollutants, degradation coefficient, water diversion discharge and diffusion coefficient. The results showed that the amount of pollutants had the most significant impact, followed by the degradation coefficient. The total phosphorus and total nitrogen concentrations of Xuanwu Lake increased with the increase of the amount of pollutants. The water quality of Xuanwu Lake decreased significantly with the increase of degradation coefficient. Increasing the water diversion discharge will not only make a big difference in water quality, but it will also worsen the water quality. The effect of the amount of pollutants on Xuanwu Lake total phosphorus and total nitrogen is 4.1 and 5.7 times that of water diversion discharge. The influence of total phosphorus and total nitrogen in the degradation coefficient scheme is 3.5 and 6.2 times that of the water diversion discharge scheme. The diffusion coefficient has almost no effect on the water quality of Xuanwu Lake. From the practical difficulty and implementation effect of water environment improvement, the order of water quality improvement effect from good to bad is as follows: the amount of pollutants scheme, degradation coefficient scheme, water diversion scheme, diffusion coefficient scheme. Under the circumstance of limited water diversion, the lake will effectively improve the water quality. Reducing the discharge of pollutants is the fundamental measure to control water environment problems, and water diversion is an auxiliary measure to improve the water ecology. It will become a trend to combine the reduction of pollutant discharge and water transfer for water environment improvement. This paper is of significance for improving the water quality of Xuanwu Lake, and it also provides a scientific method for water environment improvement of water diversion projects.

## 1. Introduction

Water is not only the origin of life, but also promotes human progress and social development. The comprehensive utilization of water resources has promoted economic development. Comprehensive utilization of water resources can be divided into non-water-consuming river use and water-consuming outside of river use. The water utilization in non-water-consuming river use includes hydropower, water transportation, fisheries, water entertainment, and other ecosystem services. The water utilization in water-consuming outside of river use includes agricultural, industrial and domestic. With the intensified development of water resources by mankind, rivers have been severely damaged, including the water environment [1,2]. Pollution of the water environment has led to the destruction of the ecological status of water, causing water bodies to lose their functions, which in turn has restricted human development. After the water environment is damaged, the ecological function of water bodies is restored by reducing pollutants discharge and water diversion [3,4]. By constructing the urban sewage pipe network, sewage enters the plant for treatment, thereby reducing direct discharge into river [5,6]. Construction of a new sewage pipe network is in conflict with the old urban infrastructure, and will also take many years to complete [7,8]. In addition, there are certain problems in the operation and management of the sewage pipe network, which makes it impossible to use reasonably, making it useless [9,10]. It is difficult to control discharge of pollutants into the river. Generally, water with better quality is diverted to avoid polluted areas through ecological water regulation to meet water quality standards [11,12]. In recent years, water diversion has gradually become a commonly used pollution control method as it can improve water quality in short time.

Water-diversion projects include the construction of manmade structure that modify the natural flow of a waterway. Diversion projects are usually developed for purposes of flood control, hydroelectric power generation, water supply, and farm irrigation. Clean freshwater is a valuable resource and is in short supply worldwide. Water diversion engineering has been successfully applied and implemented with various suitable conditions, including water diversion engineering, artificially diverting water from an existing surplus to a watershed or river to redistribute much-needed water supplies, and changing living conditions and the ecological environment [13]. There are more than 160 large-scale diversion projects built in 24 countries [14]. Lake Okeshobe reduces total phosphorous concentration by diverting the course of the Kissimmee River in the United States. In China, water diversion projects have increased in recent years. The South to North Water Diversion Project is a famous water diversion project, with a total length of 3833 km [15]. It is regarded as a strategic and ambitious solution to resolve water shortage problems in the north with three routes delivering water from the Yangtze River to the north of China [16]. The famous Great Canal from Beijing to Hangzhou with a total length of 1794 km, is the longest and first manual canal in the world [17]. The water diversion project has also been implemented in more places and has played a key role in water environment protection [18,19,20,21,22,23,24,25,26,27,28].

Mathematical models are useful scientific tools and technical means for studying water environment. The mathematical model is based on the conservation of mass, conservation of energy, and conservation of momentum, simulating the physical, chemical, and biological phenomena in water. Model calculation cannot only simulate the experiments that physical models cannot carry out, but also has the advantage of high-cost performance. Mathematical models of water environment are often used in the formulation of water resources protection and water environment treatment plans. The model is used to study the hydrodynamic characteristics, pollutants migration and transformation characteristics, so as to provide a scientific basis and theoretical support for water environment management. The water environment mathematical model consists of hydrodynamic models and water quality models. In the mid-to-late 1970s, mathematical models were used to quantitatively simulate migration, transformation, and changes in water quality. Since the 1980s, researchers have developed CREAMS (chemical runoff and erosion from agricultural management systems), ANSWERS (the areal non-point source watershed environment response simulation), and so on. In this stage, the water quality model research focused on application and gradually improved, forming the QUAL, MIKE, WASP, SWAT, OTIS, BASINS, AGNPSl, ANSWERS and EFDC model systems. The MIKE series model developed by the Danish Institute of Hydraulics is one of the most widely used commercial models, covering Mike Urban, Mike21, Mike3, Litpack, Mike Animator, Mike C-map, Eeco Lab, Mike11, Mike Flood, Feflow, Mike She, Mike Basin, and Mike21C, etc.

In recent years, despite the increase in the amount of water diversion upstream of Xuanwu Lake, its water quality has not improved. In this paper, the mathematical model of the water environment is used to study the influence of the main parameters on the water environment of Xuanwu Lake. By collecting topographic, water quality, hydrodynamic and meteorological data of Xuanwu Lake, we construct a two-dimensional mathematical model of Xuanwu Lake water environment. We obtain pollution source information through on-site investigation and sampling. Subsequently, we simulate the current water quality and use the monitoring data to verify the model so that the model can make accurate predictions. According to the current situation survey, the main factors affecting Xuanwu Lake’s water quality are as follows: the amount of pollutants (W), the degradation coefficient (K), the amount of water diversion (Q) and the diffusion coefficient (D). To investigate the effect of these four parameters on the water environment of Xuanwu Lake, we studied each parameter separately while keeping other parameters unchanged. Considering that a large quantity of outer water cannot enhance the water quality of Xuanwu Lake, we propose self-circulation measures of the lake’s water body to study whether it can improve the lake’s water quality.

## 2. Study Area and Methods

### 2.1. Study Area

**Figure 1:**
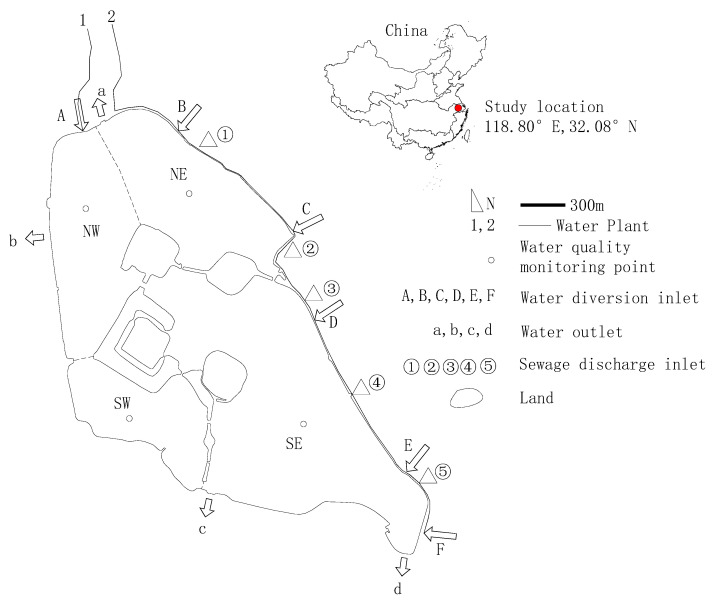
Study area.

Xuanwu Lake is a typical shallow urban lake with an aquatic area of 3.7 square kilometers and drainage area of 5.5 square kilometers. Where the surface water level is 10 m, the mean water depth is 1.14 m and the storage capacity is 4,290,000 m^3^. The highest water level is 11.15 m (Figure 1, SE), and the smallest possible surface water level is 9.8 m, annual surface water level 9.8 m to 10.2 m. The Xuanwu Lake area has a humid and warm subtropical climate. The average annual temperature is 15 °C to 16 °C. Typically, the warmest temperature is between July and August, with an average of around 28 °C. The average annual rain is around 1000 mm. The average annual wind velocity is 8 m/s, and the prevailing wind direction is southeast and northwest [4]. Since 2000, Xuanwu Lake has implemented water diversion engineering, and the water supply reached 50,000 m^3^/d. In 2003, the water supply reached to 180,000 m^3^/d, and the next year, it became 280,000 m^3^/d. The water supply for diversion comes from the Yangtze River, which is directly discharged into Xuanwu Lake through underground concealed pipes after sedimentation. There is almost no suspension of sediment. After the water is diverted through two water plants, it enters Xuanwu Lake from different locations through two pipelines, of which the No. 2 pipeline is divided into six points. The lake’s water then passes through four gates to supply water to the city’s inland rivers. The pollution sources of the lake are mainly five tributaries into the lake. Xuanwu Lake is divided into four lake areas, namely Northeast (NE) Lake, Southeast (SE) Lake, Southwest (SW) Lake and Northwest (NW) Lake. Under normal circumstances, the water supply of No. 1 water plant is 80,000 m^3^/d, and it enters Xuanwu Lake through Entrance A. The water supply of No. 2 water plant is 200,000 m^3^/d, and it enters Xuanwu Lake through the inlet BCDEF under a pipeline. The water supply to the lake through No. 3 tributary is 70,000 m^3^/d. The total amount of water transferred from Xuanwu Lake is 280,000 m^3^/d. Therefore, the total water transfer capacity of Xuanwu Lake is 350,000 m^3^/d (Figure 1). According to the water quality evaluation results of Xuanwu Lake in the past three years, the main pollutants exceeding the standard of water quality are total phosphorus and total nitrogen. Under normal water transfer conditions, the water quality concentration of Xuanwu Lake cannot meet the standard (GB 3838-2002) Class IV (TP less 0.1 mg/L, TN less 1.5 mg/L).

### 2.2. Study Methods

#### 2.2.1. Hydrodynamic Two-Dimensional Model

The two-dimensional hydrodynamic governing equations are the continuous equations and momentum equations integrated along the depth direction of the three-dimensional Reynolds Navier–Stokes average equation of incompressible fluids in the Cartesian coordinate system [29,30]. The equations are as follows:

(1) $$h\bar{u}=\int_{-d}^{\eta }udz,h\bar{v}=\int_{-d}^{\eta }vdz,\;$$

where: $\left(u_{s},v_{s}\right)$ represents the rate of discharge into the water body; $\rho_{0}$ represents the density of water.

Continuity equation:

(2) $$\frac{\begin{array}{c} \partial h \end{array}}{\partial t}+\frac{\partial h\bar{u}}{\partial x}+\frac{\partial h\bar{v}}{\partial y}=hQ\;$$

Momentum equation:

(3) $$\frac{\partial h\bar{u}}{\partial t}+\frac{\partial h{\bar{u}}^{2}}{\partial x}+\frac{\partial h\bar{vu}}{\partial y}=fh\bar{v}-gh\frac{\partial \eta }{\partial x}-\frac{h}{\rho_{0}}\frac{\partial P_{a}}{\partial x}-\frac{gh^{2}}{2\rho_{0}}\frac{\partial \rho }{\partial x}+\frac{\tau_{sx}}{\rho_{0}}-\frac{\tau_{bx}}{\rho_{0}}-\frac{1}{\rho_{0}}\left(\frac{\partial S_{xx}}{\partial x}+\frac{\partial S_{xy}}{\partial y}\right)+\frac{\partial }{\partial x}\left(hT_{xx}\right)+\frac{\partial }{\partial y}\left(hT_{xy}\right)+hu_{s}Q$$

(4) $$\frac{\partial h\bar{v}}{\partial t}+\frac{\partial h{\bar{v}}^{2}}{\partial y}+\frac{\partial h\bar{vu}}{\partial x}=-fh\bar{u}-gh\frac{\partial \eta }{\partial y}-\frac{h}{\rho_{0}}\frac{\partial P_{a}}{\partial y}-\frac{gh^{2}}{2\rho_{0}}\frac{\partial \rho }{\partial y}+\frac{\tau_{sy}}{\rho_{0}}-\frac{\tau_{by}}{\rho_{0}}-\frac{1}{\rho_{0}}\left(\frac{\partial S_{yx}}{\partial x}+\frac{\partial S_{yy}}{\partial y}\right)+\frac{\partial }{\partial x}\left(hT_{xy}\right)+\frac{\partial }{\partial y}\left(hT_{yy}\right)+hv_{s}Q$$

where: *x*, *y* are Cartesian coordinates; *t* is time; *h* is total water depth; *η* is water level;$\;\bar{u}$ and $\bar{v}$ are average water depth; *ρ* is water density; *f* = 2*Ωsinφ* represents the Coriolis factor (*φ* is the geographical latitude *Ω*, is the angular velocity of the Earth’s rotation); $S_{xx}$, $S_{xy}$ and $S_{yy}$ represent the radiation stress tensors; *Q* represents the point source emissions; g represents the gravitational acceleration; and *P_a_* represents the atmospheric pressure.

Transverse stress *T_ij_*, involves viscous resistance, dynamic advection friction resistance, and turbulent frictional resistance, which can be calculated using the vortical viscosity equation for average vertical velocity:

(5) $$T_{xx}=2A\frac{\partial \bar{u}}{\partial x},\;T_{xy}=A\left(\frac{\partial \bar{u}}{\partial y}+\frac{\partial \bar{v}}{\partial x}\right),\;T_{yy}=2A\frac{\partial \bar{v}}{\partial x}.$$

#### 2.2.2. Two-Dimensional Water Quality Model

The water quality equations are based on the mass balance equation. The model that meets the calculation purpose must be two-dimensional or three-dimensional, while considering that the data is suitable for a two-dimensional model. The results of two-dimensional calibration can reflect the actual situation of hydrodynamic and water quality [31,32]. The two-dimensional water quality transport equation is as follows:

(6) $$\frac{\partial C_{i}}{\partial t}+U\frac{\partial C_{i}}{\partial x}+V\frac{\partial C_{i}}{\partial y}=\frac{\partial }{\partial x}\left(E_{x}\frac{\partial C_{i}}{\partial x}\right)+\frac{\partial }{\partial y}\left(E_{y}\frac{\partial C_{i}}{\partial y}\right)+K_{i}C_{i}+S_{i},$$

where: *C_i_* represents the water pollutants concentration; *E_x_* and *E_y_* are the diffusion coefficients in the *x* and *y* axes, respectively; *u*, *v* are the flow velocity components in the *x* and *y* axes, respectively; *K_i_* is the pollutant degradation coefficient; and *S_i_* represents the pollutant sediment release coefficient.

### 2.3. Model Setup and Calibration

#### 2.3.1. Model Setup

**Figure 2:**
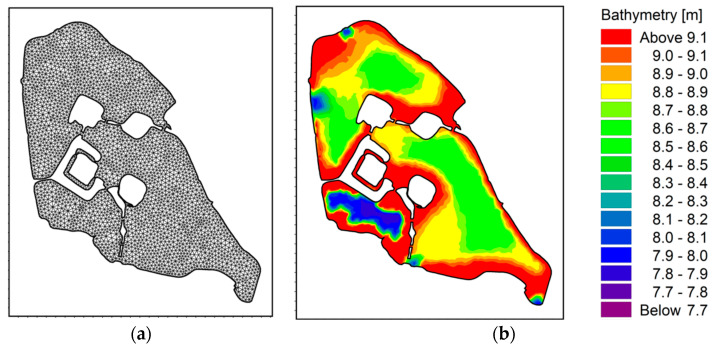
(**a**) Xuanwu Lake model grid. (**b**) Bottom elevation.

The Xuanwu Lake model is subdivided into a mixed network of three quadrants of angles with mesh spacing from about 20 to 30 m [11,17]. Assuming that the lake surface is stationary at the initial moment, there is no disturbance, and the time step *t* = 1 d [29,30]. The grid and topography of the Xuanwu Lake model are shown in Figure 2. The depth at the floor of the bottom of Xuanwu Lake ranges from 0.8 m to 2.2 m, the deepest area is Southwest Lake, and the deepest water depth is 2.2 m [33,34]. According to the measured topography of the wading area of Xuanwu Lake and the bottom elevation of the model, through model verification, the lake’s Manning coefficient is 38 m^1/3^/s, and the eddy current viscosity is 0.28 [35,36].

#### 2.3.2. Boundary Conditions

**Figure 3:**
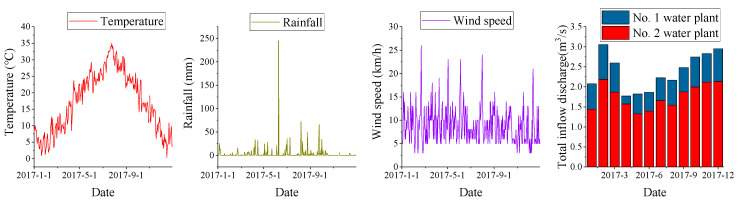
Daily temperature, rainfall, wind speed, and monthly total inflow discharge of Xuanwu Lake in 2017.

Hydrodynamic parameters: the temperature is 10 °C, the initial water level is 10 m, and the flow rate is 0 at the start [9,10,37]. The water level of the gate at the outlets a, b, c, d is 3 m. The temperature, wind velocity, rainfall and average monthly inflow rate for 2017 are shown in Figure 3. The meteorological data source comes from website (http://data.sheshiyuanyi.com/WeatherData/, accessed on February 2021) and water plant.

**Figure 4:**
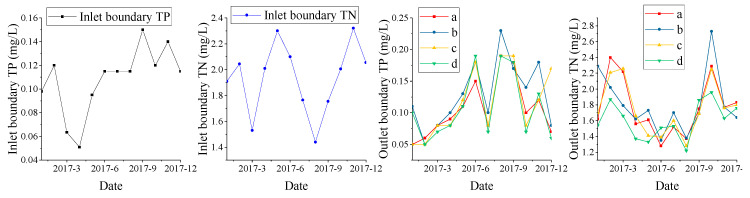
Inlet and outlet boundary water quality of Xuanwu Lake in 2017.

Water quality parameters: the pollutant (TP, TN) concentration of the inflow and of the boundary of the a, b, c, and d outlets are as shown in Figure 4. Water quality data were obtained from the Nanjing Environmental Protection Bureau.

#### 2.3.3. Model Calibration

**Figure 5:**
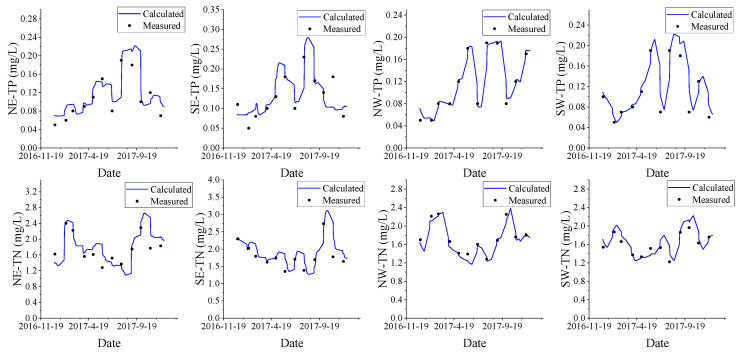
Model (water quality) validation of Xuanwu Lake in 2017.

This paper uses a trial-and-error method to calibrate and verify the model parameters. According to the calculation of the 2D water quality model [38,39,40], the TN degradation coefficient K of Xuanwu Lake was 8.55 × 10^−7^ s^−1^ to 1.41 × 10^−6^ s^−1^, and the TP degradation coefficient K was 5.92 × 10^−7^ s^−1^ to2.26 × 10^−6^ s^−1^. The pollutants (TP, TN) concentration calibration and verification in the districts of NE, SE, NW, SW are shown in Figure 5. Table 1 shows that the error between measured and calculated data of water pollutants in the whole lake of different months were less than 20%. It can be seen that the model can be used for model simulation and prediction [41,42,43].

### 2.4. Calculation Schemes

To study the relationship between water quality and the influencing factors of Xuanwu Lake, we set different schemes for the amount of pollutants (W), degradation coefficient (K), water diversion (Q) and diffusion coefficient (D). Based on the current amount of pollutants and reduction plan, 11 schemes were set up to calculate the amount of pollutants after reduction [7,44], and the relationship between the amount of pollutants and the water quality of Xuanwu Lake was studied (Table S1). Based on the current range of degradation coefficients, 27 schemes were set up to calculate the water quality under different degradation coefficients [45], and the relationship between water pollutants concentration and degradation coefficients of Xuanwu Lake was shown in Table S2. Based on the water diversion capacity of 350,000 m^3^/d and current actual amount of diversion, 16 types of water diversion schemes were calculated, and the relationship between the water pollutant concentration and water diversion of Xuanwu Lake was studied (Table S3). We also studied 19 kinds of diffusion coefficient schemes (Table S4). The schemes and results are presented in Table S4. We calculated the total phosphorus (TP) and total nitrogen (TN) of the southeast (SE), northeast (NE), southwest (SW), northwest (NW), and the whole lake (Whole).

## 3. Results and Discussion

### 3.1. Impact of Pollutant Quantity (W) on Water Quality

**Figure 6:**
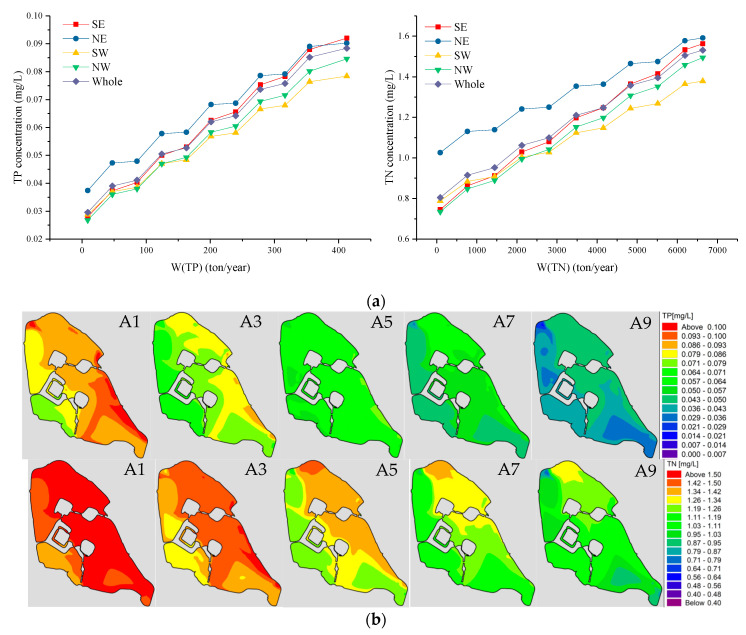
(**a**) Water quality trend of various lake areas in Xuanwu Lake with the discharge of pollutants. (**b**) The first row shows the TP concentration (mg/L) fields from left to right for Schemes A1, A3, A5, A7, and A9; the second row shows the TN concentration (mg/L) fields from left to right for Schemes A1, A3, A5, A7, and A9.

Keeping other conditions unchanged, the water quality change of Xuanwu Lake is calculated by reducing the emission concentration of pollutants. It can be seen from Figure 6a that the TP and TN concentrations of the lake increase with the increase in the amount of pollutants. Due to the order of magnitude difference in the concentration of TN and TP, the average concentration of nutrients is dominated by TN. According to routine monitoring of water quality data, the average concentration of water pollutants of the Northeast Lake is the highest of the four lake regions, and the average water quality of the Southwest Lake is the lowest. The pollutant concentration in the Southeast Lake area is close to that of the whole lake. The overall order of water quality from good to bad is Southwest Lake, Northwest Lake, Southeast Lake, Northeast Lake. As shown in Figure 1 and Figure 6a,b, the main reason why the pollutants concentration of the western lake area is better than the eastern lake area is that pollutants are mainly discharged from the eastern half of the lake. The maximum pollutant emission plan is the status quo. The concentration of TP is 0.0884 mg/L (less than the standard value of 0.1 mg/L), and the water quality of TN is 1.53 mg/L (more than the standard value of 1.5 mg/L). As long as there is one item that does not meet the standard, the conclusion is that it does not meet the standard overall. After reducing TN pollutants by 6.7%, TN was just able to meet the standard. When there are no pollutant discharged into Xuanwu Lake, and only non-point source pollution exists, the TP and the TN concentration are 0.03 mg/Land 0.81 mg/L, respectively.

Based on the data analysis results, we calculated the concentration fields of scheme A1, A3, A5, A7, and A9 (Table S1), respectively, as shown in Figure 6b. The red area in the figure represents the area where the water quality is not up to standard. It can be seen that as the amount of pollutants decreases, the high-concentration of water quality gradually decreases and the low-concentration area gradually increases. Although the amount of TP and TN pollutants is reduced by the same proportion, the concentration of TP can meet the standard more easily than TN.

### 3.2. Impact of Degradation Coefficient (K) on Water Quality

**Figure 7:**
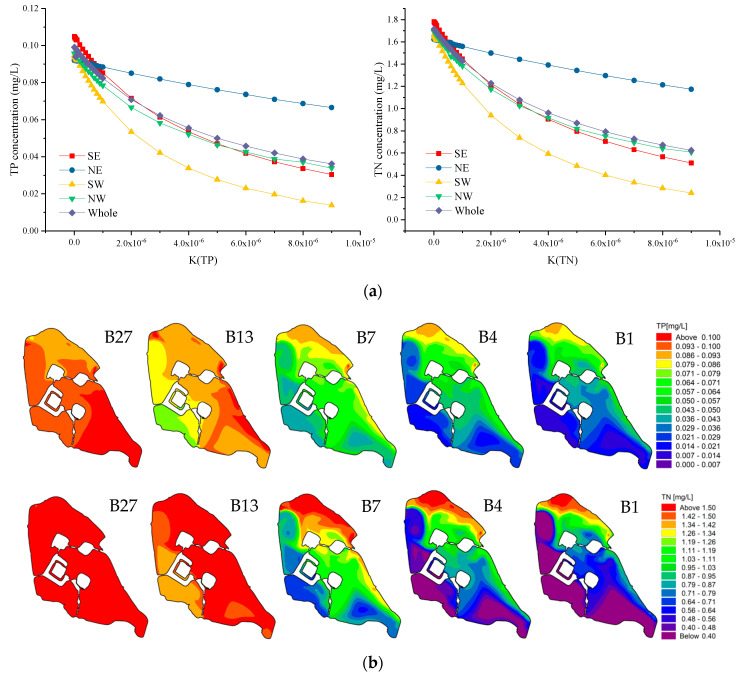
(**a**) Water quality trend of various areas in Xuanwu Lake with the degradation coefficient (K). (**b**) The first row shows the TP concentration (mg/L) fields from left to right for Schemes B27, B13, B7, B4, and B1; the second row shows the TN concentration (mg/L) fields from left to right for Schemes B27, B13, B7, B4, and B1.

We calculate the reasonable degradation coefficient [4] schemes of Xuanwu Lake while keeping other conditions unchanged. Within the degradation coefficient of Xuanwu Lake, the change range of TP was 0.036~0.099 mg/L, and the change range of TN was 0.62 to 1.72 mg/L, as shown in Figure 7a. On the whole, the water pollutants concentration of Xuanwu Lake decreased significantly with the increase in the degradation coefficient. The concentration trend of water quality in all lake areas is the same, and the rate of concentration decline gradually decreases. However, Southwest Lake with the best water quality has the largest change trend, followed by Southeast Lake, then Northwest Lake and Northeast Lake. The pollutant concentrations of the lake areas (K_TP_ < 1 × 10^−6^, K_TN_ < 5 × 10^−7^), from large to small are Southeast Lake, Northeast Lake, Northwest Lake, and Southwest Lake. When the degradation coefficient K is less than 0.000008 d^−1^, the water quality of Northeast Lake is greater than that of Southeast Lake. The lake area with the largest decline rate of pollutant concentration has the smallest pollutant concentration. In general, the pollutant concentration of Southeast Lake and Northwest Lake are essentially the same, which is the same as that of the whole lake. That is, the water quality of Southeast Lake and Northwest Lake can represent the average water quality of the whole lake. When the degradation coefficient is very small, the water quality of the four lakes is essentially the same. With the increase of degradation coefficient, the pollutant concentration change rate of Southeast Lake and Northwest Lake is essentially the same, which the difference between the pollutant concentration of the Northeast Lake Area and the Southwest Lake Area becomes larger, and the pollutant concentration difference from other lake areas becomes larger. 

We calculated the concentration fields for the B27, B13, B7, B4, and B1 schemes (Table S2), as shown in Figure 7b. The TP of each scheme can meet water quality standard (GB 3838-2002) Class IV, while the TN of B27 and B13 schemes cannot. Red represents areas where the water quality is not up to standard. It can also be seen in Figure 7b that as the degradation coefficient increases, the red area gradually decreases, the blue area gradually increases, and the water quality improves. It can be seen that the water pollutants concentration of each lake area changes asynchronously with the degradation coefficient. With the increase in the degradation coefficient, the order from greatest to lowest of the lake areas that have improved water quality is Southwest Lake, Northwest Lake, Southeast Lake, and Northeast Lake. 

### 3.3. Impact of Discharge (Q) on Water Quality

**Figure 8:**
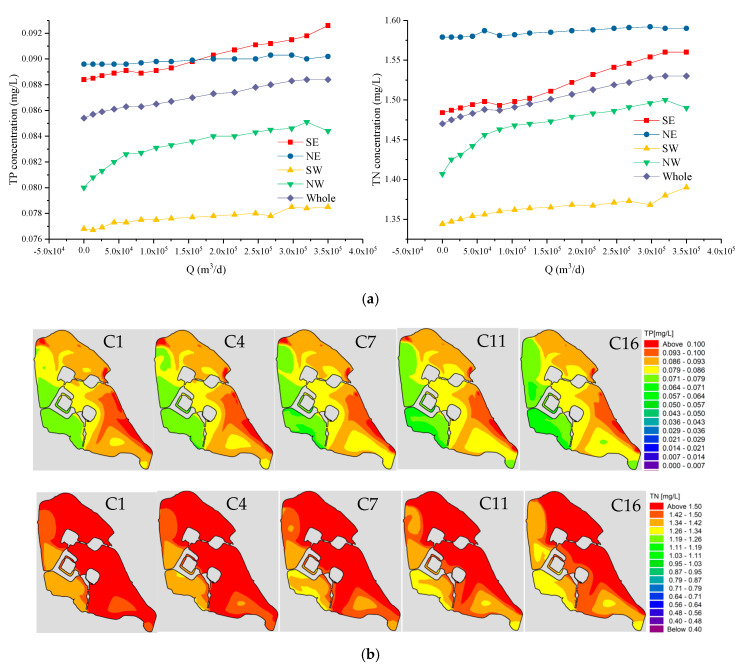
(**a**) Water quality trend of various lake areas in Xuanwu Lake with the discharge (Q). (**b**) The first row shows the TP concentration (mg/L) fields from left to right for Schemes C1, C4, C7, C11, and C16; the second row shows the TN concentration (mg/L) fields from left to right for Schemes C1, C4, C7, C11, and C16.

Under the condition that the water diversion concentration is kept constant, reasonable water diversion schemes are calculated to obtain the water concentration of Xuanwu Lake, as shown in Figure 8a. The variation range of TP concentration in each lake area is 0.0767 to 0.0926 mg/L, and the variation range of TN is 1.344 to 1.592 mg/L. The variation interval of TP and TN concentration of the pollutant amount (W) schemes is 4.1 and 5.7 times that of the concentration variation range of this scheme. The variation interval of TP and TN concentration of the degradation coefficient (K) schemes is 3.5 and 6.2 times that of the concentration variation range of this scheme. With the increase in diversion flow, the pollutant concentration of each lake area increased slightly. The order of growth rate of pollutant concentration in each lake area from large to small is Southeast Lake, Northwest Lake, Southwest Lake, Northeast Lake. In this scheme, the order from large to small of the overall water concentration for each lake is, Northeast Lake, Southeast Lake, Northwest Lake, and Southwest Lake. However, after the diversion flow became greater than 150,000 m^3^/d, the TP concentration in Southeast Lake was greater than the TN concentration in Northeast Lake. We compare this scheme with the pollutant quantity scheme (K). Plan A5 and Plan C16 add the same amount of TP and TN into the river. We compare this scheme with the pollutant quantity scheme (K). In the case of 350,000 m^3^/d water diversion, the concentrations of TP and TN are 0.062 mg/L and 1.1 mg/L, respectively. Without water diversion, the concentrations of TP and TN are 0.0854 mg/L and 1.47 mg/L, respectively.

We calculated the TP and TN concentration fields for the C1, C4, C7, C11, and C16 schemes (Table S3, Figure 8b). The results reveal that with the increase in water diversion, the concentration field in each lake area changes little. As with other schemes, the pollutant concentration in the western district of lake is better than the left lake area. It can be seen from the figure that the area exceeding the TP standard is small, and most of the lake areas can meet the standard. However, the area exceeding the TN standard is very large, and most of the lake areas cannot meet the standard. This phenomenon is consistent with the results of 16 calculation schemes.

### 3.4. Impact of Diffusion Coefficient (D) on Water Quality

**Figure 9:**
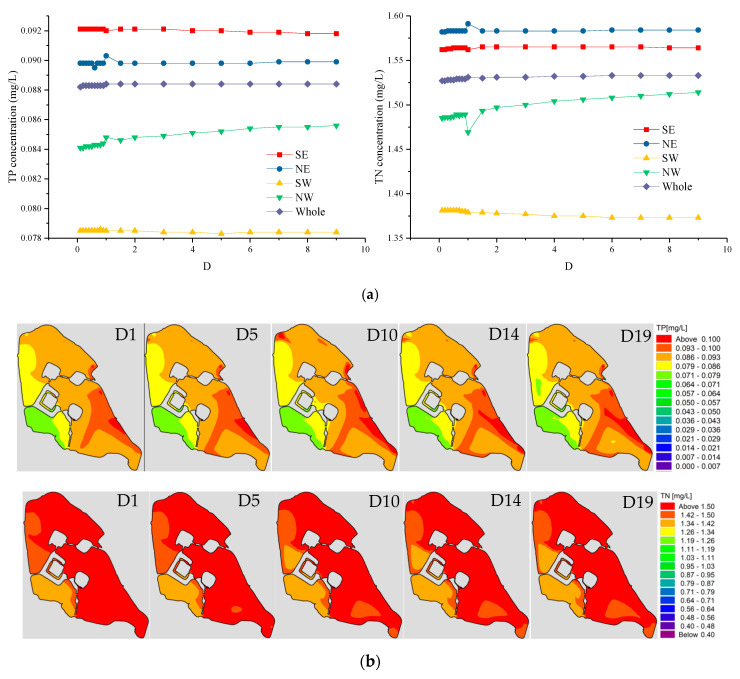
(**a**) Water quality trend of various lake areas in Xuanwu Lake with the diffusion coefficient (D). (**b**) The first row shows the TP concentration (mg/L) fields from left to right for Schemes D1, D5, D10, D14, and D19; the second row shows the TN concentration (mg/L) fields from left to right for Schemes D1, D5, D10, D14, and D19.

We calculated the water concentration of Xuanwu Lake under different diffusion coefficient schemes while keeping other conditions unchanged. The diffusion coefficient of water pollutants is mainly affected by temperature and velocity [4]. When the value of the diffusion coefficient ranges from 0.1 to 9, the pollutants concentration of each lake area does not change significantly. However, with the increase in the diffusion coefficient, there was a slight increase in water quality in the Northwest Lake area, where TP increased by 0.0015 mg/L and TN increased by 0.02 mg/L. Such small changes are negligible for current pollutant concentrations. We calculated the TP and TN concentration fields for the D1, D5, D10, D14, and D19 schemes (Table S4), respectively, as shown in Figure 9b. It can be seen from the figure that under different diffusion coefficients, the concentration field in each lake area is almost the same. 

### 3.5. Analysis of Lake Water Body Self-Circulation

According to previous calculations, Southwest Lake has the lowest pollutants concentration and the best water quality, this is due to its low mobility, which makes it is difficult for pollutants to reach Southwest Lake. With the consideration of saving water resources and energy, diversion water from Southwest Lake to Northwest Lake will greatly improve the self-circulation of the lake’s water body. The transfer of water from Southwest Lake to Northwest Lake is conducive to improving the uniformity of water quality, thereby improving water quality. Under certain lake diversion flow (350,000 m^3^/d), as the self-circulating flow (1 m^3^/s, 2 m^3^/s, 3 m^3^/s, 4 m^3^/s, 5 m^3^/s) in the lake increases, the water pollutant concentration in each lake area decreases. However, the pollutants concentration in the East Lake area is still higher than in other lake areas. The water quality intervals are the same. The water quality in the East Lake area is the same as other situations, but the water concentration in the West Lake area decreases significantly with the increase in water flow in the lake. The results show that under the condition of water diversion of 350,000 m^3^/d, the water quality of the condition of 5 m^3^/s self-circulation is the best. Without water self-circulation in the lake, the pollutant concentration in the lake tends to increase with the increase of the water flow. However, with the increase in self-circulation flow, water quality has improved significantly, especially in the West Lake area, but water quality of the Northeast Lake did not change with the increase in self-circulation flow. The flow field is virtually unchanged under different self-circulating flows. There is only a slight change in the bridge hole. Generally, the local flow velocity increases with the increase in the amount of water diversion.

### 3.6. Discussion

The two-dimensional mathematical model of water environment used in this paper to study the lake water environment can meet the purpose of calculation entirely and reflect the real situation of hydrodynamic and water quality. The equation discrete method of the model is solved by the explicit finite volume method at the center of the unit, which ensures the conservation of water and momentum in the calculation domain. The model uses unstructured triangle or quadrilateral grid elements, which is more conducive to fitting complex boundary lines. It is convenient and quick to use the wet and dry grid judgment method to deal with the moving boundary of the tidal flat. Use of parallel computing can greatly reduce the computing time. The model is suitable for simulating wide rivers, lakes, and oceans, but it is not suitable for narrow rivers because of its difficulty in dividing the grid.

Keeping other conditions unchanged, the TP and TN concentrations of Xuanwu Lake increase with the increased amount of pollutants. The TP concentration range was 0.0268 to 0.092 mg/L, and the TN concentration range was 0.735 to 1.59 mg/L. The overall order of water quality from good to bad is Southwest Lake, Northwest Lake, Southeast Lake, Northeast Lake. Reducing pollutant emissions is the fundamental way to improve water quality. The reduction of pollutants entering Xuanwu Lake is mainly achieved by reducing the direct discharge of domestic sewage from surrounding residents. For areas without a sewage pipe network, the construction of the pipe network should be strengthened to connect sewage to a sewage treatment plant. At the same time, lake silt dredging needs to be carried out regularly due to the impact of sediment on lake water quality in the future. In contrast, the most important factor affecting the water environment of an artificial lake is the amount of pollutants discharged. Controlling the discharge of pollutants controls the quality of the water environment. The pollutants of lakes in nature change very little, and only human factors cause water pollution. Surrounding Xuanwu Lake are urban residents. Domestic sewage is discharged into the lake through the river channel. The interference of humans increases the pollutants in the lake and causes the deterioration of water quality. The Funil Reservoir in Brazil [46] and Lake Okeechobee [47] in the United States are affected by human agriculture, and the water quality changes seasonally. Xuanwu Lake is different from the natural lake Loktak Lake, and is the same as Funil Reservoir and Lake Okeechobee, which are also interfered by humans.

In the calculation of the degradation coefficient schemes, the change range of TP was 0.036 to 0.099 mg/L, and the change range of TN was 0.62 to 1.72 mg/L, within the suitable degradation coefficient of Xuanwu Lake. The water quality of Xuanwu Lake decreased significantly with the increase in the degradation coefficient. The pollutants concentrations from large to small lake areas are, Southeast Lake, Northeast Lake, Northwest Lake, and Southwest Lake. The order in value of the decline rate of pollutant concentration is completely opposite to that of the pollutant concentration. The water quality of Southeast Lake and Northwest Lake can represent the water quality of the whole lake, allowing the reduction of the monitoring frequency of lake water quality. If the degradation coefficient is adjusted by human interference, the water quality of Xuanwu Lake can also be improved. The degradation coefficient of water body can be improved by spreading degrading bacteria, rationally adjusting water, widening river channels, and dredging silt. In the calculation of the diffusion coefficient schemes, when the value of the diffusion coefficient ranges from 0.1 to 9, the pollutant concentration of each lake area does not change significantly. However, with the increase in the diffusion coefficient, there was a slight increase in water quality in the Northwest Lake area; TP concentration increased by 0.0015 mg/L and TN concentration increased by 0.02 mg/L. The main factors affecting the diffusion coefficient are temperature and flow rate. In the water quality parameter study of Igapó I Lake (Londrina, Paraná, Brazil) [48], Shagawa Lake (MN, USA) [49], and Koumoundourou Lake (brackish lake, Eleusis Bay), the range of degradation coefficient and diffusion coefficient were also studied. Xuanwu Lake measurements show differences when compared with the degradation coefficients and diffusion coefficients of lakes in different parts of the world, but they all have a range of values. It can be seen from the comparison of the data that the water quality cannot be improved quickly by only increasing the water diversion. From the perspective of improving water quality, reducing pollutants is more effective than water transfer. It is more time-efficient to improve water quality through joint application to reduce some pollutants and water diversion.

In the calculation of water diversion schemes, the change range of TP concentration in each lake area is 0.0767 to 0.0926 mg/L, and the change range of TN is 1.344 to 1.592 mg/L. With the increase in water diversion flow, the pollutant concentration of each lake area increased slightly. The number of pollutant schemes affected the TP and TN concentration of Xuanwu Lake by 4.1 and 5.7 times as much as the discharge schemes. By increasing the degradation coefficient, the TP and TN concentrations in Xuanwu Lake were reduced by 3.5 and 6.2 times that of the water diversion schemes. The water quality of Xuanwu Lake cannot be improved by only increasing the diversion water, and doing so would reduce the water quality and cause unnecessary waste of resources. This paper takes the discharge of pollutants during the dry season as an example; combined with the ecological protection needs of Xuanwu Lake, its suitable water diversion flow is 180,000 m^3^/d. With the self-circulation flow, the water quality of Xuanwu Lake has improved significantly, especially under the condition of 350,000 m^3^/d. With the increase in self-circulation flow, the water quality of Xuanwu Lake will also improve; however, the flow field in the lake area has not changed significantly thus far. It can be seen that the lake self-circulation can be implemented when the water diversion capacity of the lake is limited or when the water quality cannot be changed with water diversion. Lake self-circulation can not only improve the water quality of the lake, but also avoid damage to the lake’s flow field. Based on the research of this paper, measures to improve the water quality of Xuanwu Lake are proposed. Reducing the discharge of pollutants is the most fundamental measure to improve the quality of the water environment. Improving the quality of the diversion water will also improve the water environment of Xuanwu Lake to a certain extent. It is recommended to open up Southwest Lake and Northwest Lake to make the water flow self-circulate in the lake area, thereby improving water quality. It is recommended to construct a water pump with a volume of one cubic meter in the Southwest Lake, so that the water in the Southwest Lake can be transferred to the Northwest Lake, and the water can be self-circulated, thereby improving the water quality.

Based on the real data collected in Xuanwu Lake, the research method of mathematical modelling was used to determine the factors influencing changes of water quality parameters. The MIKE21 mathematical model has been widely used all over the world, with theoretical and practical foundations, and reliability. The research results of this paper are also applicable to small lakes with a water exchange cycle of one week, which has certain promotional value. The limitation of this study is that it is not applicable to large shallow lakes, such as Taihu Lake (China) and Lake Okeechobee (USA). Xuanwu Lake is different from large lakes and lakes in nature. Generally, it is small artificial lakes that exist in fast-growing cities. The main issues of small lakes addressed in this paper are the amount of pollutants, degradation coefficient, water diversion, and diffusion coefficient, and does not involve the problem of cyanobacteria blooms and sediment resuspension. This paper has certain reference value for the water environment management of small lakes.

With the development of global urbanization and the increase in human demand for water landscapes, there will be more and more small artificial lakes similar to Xuanwu Lake. The water environment of the lake will gradually deteriorate with human intervention. The study found that reducing the discharge of pollutants is the key to improving the water quality of small artificial lakes. In the long term, due to the gradual implementation of the construction of the sewage pipe network, pollutants are gradually discharged into the sewage plant, which gradually improves the lake water quality. Appropriate aeration reoxygenation, artificial hydraulic circulation and diafiltration purification can change the degradation coefficient and diffusion coefficient, and can also improve water quality.

## 4. Conclusions

Aimed at the fact that the pollutants concentration of Xuanwu Lake cannot reach the standard stably, we have carried out research on factors influencing water quality with the two-dimensional water environment mathematical model. The effects of pollutants quantity (W), degradation coefficient (K), water diversion (Q), and diffusion coefficient (D) on the water quality of Xuanwu Lake were studied. The purpose was to find out the influence of factors on the water quality of Xuanwu Lake, and to propose water quality improvement measures. The main conclusions are as follows:

(1) Keeping other conditions unchanged, the TP and TN concentrations of Xuanwu Lake increase with the increase in the amount of pollutants. The TP concentration range was 0.0268~0.092 mg/L, and the TN concentration range was 0.735~1.59 mg/L. The overall order of water quality from low to high is Southwest Lake District, Northwest Lake District, Southeast Lake District, and Northeast Lake District. Reducing pollutants emissions is the fundamental way to improve water quality. The reduction of pollutants entering Xuanwu Lake is mainly achieved by reducing the direct discharge of domestic sewage from surrounding residents. For areas without a sewage pipe network, the construction of the pipe network should be strengthened so that sewage can be connected to a sewage treatment plant for treatment. At the same time, lake silt dredging needs to be carried out regularly to reduce the impact of sediment on lake water quality.
(2) In the calculation of the degradation coefficient scheme, within the degradation coefficient of Xuanwu Lake, the change range of TP was 0.036~0.099 mg/L, and the change range of TN was 0.62~1.72 mg/L. The water quality of Xuanwu Lake decreased significantly with the increase of degradation coefficient. The water quality concentrations from large to small lake areas are Southeast Lake, Northeast Lake, Northwest Lake, and Southwest Lake. The rate of decline of water quality concentration is completely opposite to that of water quality concentration. The water quality of Southeast Lake and Northwest Lake can represent the water quality of the whole lake, which can reduce the monitoring frequency of lake water quality. If the degradation coefficient is adjusted by human interference, the water quality of Xuanwu Lake can also be improved.
(3) In the calculation of the water diversion scheme, the variation range of TP concentration in each lake area is 0.0767~0.0926 mg/L, and the variation range of TN is 1.344~1.592 mg/L. With the increase in the diversion flow, the water quality concentration of each lake area increased slightly. The reduction in Xuanwu Lake concentration by reducing TP and TN was 4.1 and 5.7 times that of the water diversion plan. By increasing the degradation coefficient, the TP and TN concentrations in Xuanwu Lake were reduced by 3.5 and 6.2 times that of the water diversion plan. The water quality of Xuanwu Lake cannot be improved by only increasing the replenishment flow, and doing so would reduce the water quality and cause unnecessary waste of resources. This paper takes the discharge of pollutants during the dry season as an example, and combined with the ecological protection needs of Xuanwu Lake, its suitable replenishment flow is 180,000 m^3^/d.
(4) In the calculation of the diffusion coefficient scheme, when the value of the diffusion coefficient ranges from 0.1 to 9, the water quality concentration of each lake area does not change significantly. However, with the increase in the diffusion coefficient, there was a slight increase in water quality in the Northwest Lake area, where TP increased by 0.0015 mg/L and TN increased by 0.02 mg/L. The main factors affecting the diffusion coefficient are temperature and flow rate. The increase in temperature makes the water body diffuse faster, and the temperature has no influence on the water quality.
(5) With the self-circulation flow, the water quality of Xuanwu Lake has improved significantly, especially under the condition of 350,000 m^3^/d. With the increase in self-circulation flow, the water quality of Xuanwu Lake will also improve, but the flow field in the lake area has not changed significantly thus far. It can be seen that the lake self-circulation can be implemented when the water diversion capacity of the lake is limited or the water quality cannot be changed with water diversion. Lake self-circulation can not only improve the water quality of the lake, but also avoids damage to the lake’s flow field.

## Figures and Tables

**Table 1:** Error analysis table.

Time	TP				TN			
	K (s^−1^)	Simulation (mg/L)	Measured (mg/L)	Error (%)	K (s^−1^)	Simulation (mg/L)	Measured (mg/L)	Error (%)
2017-1-3	1.53 × 10^−6^	0.0811	0.0775	4.65	1.1 × 10^−6^	1.7385	1.7875	2.74
2017-2-7	2.26 × 10^−6^	0.0608	0.0525	15.81	9.26 × 10^−7^	2.1463	2.125	1.00
2017-3-1	1.53 × 10^−6^	0.0793	0.0775	2.39	9.74 × 10^−7^	2.1613	1.9825	9.02
2017-4-6	1.35 × 10^−6^	0.0929	0.0875	6.14	1.27 × 10^−6^	1.5486	1.5525	0.25
2017-5-4	1.01 × 10^−6^	0.1269	0.1175	7.99	1.29 × 10^−6^	1.5589	1.52	2.56
2017-6-2	6.77 × 10^−7^	0.1713	0.175	2.10	1.41 × 10^−6^	1.5040	1.3825	8.79
2017-7-3	1.44 × 10^−6^	0.0988	0.0825	19.70	1.24 × 10^−6^	1.5623	1.5875	1.58
2017-8-1	5.92 × 10^−7^	0.1805	0.2	9.74	1.47 × 10^−6^	1.5150	1.3125	15.43
2017-9-4	6.58 × 10^−7^	0.1980	0.18	10.01	1.13 × 10^−6^	1.7373	1.7475	0.58
2017-10-2	1.21 × 10^−6^	0.1125	0.0975	15.39	8.55 × 10^−7^	2.3060	2.3075	0.07
2017-11-1	8.61 × 10^−7^	0.1141	0.1375	17.05	1.1 × 10^−6^	1.9879	1.7325	14.74
2017-12-4	1.25 × 10^−6^	0.1113	0.095	17.20	1.1 × 10^−6^	1.9409	1.7575	10.44

## Data Availability

Not applicable.

## Supplementary Materials

The following are available online at https://www.mdpi.com/article/10.3390/ijerph18115757/s1, Table S1: Calculation Programs and Results (W), Table S2: Calculation Programs and Results (K), Table S3: Calculation Programs and Results (Q), Table S4: Calculation Programs and Results (D).
