# Supplementary material for: Simulation of the Parameters Effecting the Water Quality Evolution of Xuanwu Lake, China
Source: Int J Environ Res Public Health. 2021 May 27;18(11):5757. doi: 10.3390/ijerph18115757 (PMC8198726; doi:10.3390/ijerph18115757)
Supplement: Supplementary file 1 [file ijerph-18-05757-s001.zip › ijerph-1194697-supplementary.pdf]

International Journal of Environmental Research and Public Health

MDPI

Table S1. Calculation Programs and Results (W).

Schemes			TP Concentration (mg/L)					TN Concentration (mg/L)				
Plan	W (ton/year)		SE	NE	SW	NW	Whole	SE	NE	SW	NW	Whole
	TP	TN										
A1	412.7	6635.9	0.0920	0.0902	0.0784	0.0846	0.0884	1.56	1.59	1.38	1.49	1.53
A2	354.9	6190.6	0.0879	0.0890	0.0764	0.0802	0.0852	1.53	1.58	1.36	1.46	1.50
A3	316.5	5512.9	0.0783	0.0792	0.0679	0.0716	0.0758	1.41	1.47	1.27	1.35	1.39
A4	278.1	4835.1	0.0753	0.0786	0.0666	0.0693	0.0736	1.36	1.47	1.24	1.31	1.36
A5	239.7	4157.4	0.0656	0.0687	0.0581	0.0605	0.0642	1.25	1.36	1.15	1.20	1.25
A6	201.2	3479.6	0.0626	0.0682	0.0568	0.0583	0.0620	1.20	1.35	1.12	1.15	1.21
A7	162.8	2801.8	0.0530	0.0583	0.0484	0.0493	0.0527	1.08	1.25	1.03	1.04	1.10
A8	124.4	2124.1	0.0500	0.0578	0.0470	0.0470	0.0505	1.03	1.24	1.00	0.99	1.06
A9	85.9	1446.3	0.0403	0.0479	0.0386	0.0380	0.0411	0.91	1.14	0.91	0.89	0.95
A10	47.5	768.6	0.0373	0.0473	0.0370	0.0360	0.0390	0.86	1.13	0.88	0.85	0.92
A11	9.1	90.8	0.0280	0.0374	0.0288	0.0268	0.0296	0.75	1.03	0.79	0.74	0.81

Table S2. Calculation Programs and Results (K).

Schemes			TP Concentration (mg/L)					TN Concentration (mg/L)				
Plan	K (d^{-1})		SE	NE	SW	NW	Whole	SE	NE	SW	NW	Whole
	TP	TN										
B1	9×10^{-6}	9×10^{-6}	0.0304	0.0665	0.0138	0.0338	0.0361	0.51	1.17	0.24	0.61	0.62
B2	8×10^{-6}	8×10^{-6}	0.0336	0.0687	0.0162	0.0370	0.0388	0.57	1.21	0.28	0.64	0.67
B3	7×10^{-6}	7×10^{-6}	0.0373	0.0710	0.0197	0.0388	0.0420	0.63	1.25	0.34	0.69	0.73
B4	6×10^{-6}	6×10^{-6}	0.0418	0.0736	0.0230	0.0426	0.0457	0.71	1.30	0.40	0.75	0.79
B5	5×10^{-6}	5×10^{-6}	0.0470	0.0761	0.0277	0.0464	0.0500	0.79	1.34	0.48	0.82	0.87
B6	4×10^{-6}	4×10^{-6}	0.0534	0.0789	0.0338	0.0521	0.0555	0.90	1.39	0.59	0.92	0.96
B7	3×10^{-6}	3×10^{-6}	0.0613	0.0819	0.0421	0.0582	0.0622	1.04	1.44	0.74	1.03	1.08
B8	2×10^{-6}	2×10^{-6}	0.0715	0.0850	0.0534	0.0666	0.0708	1.21	1.50	0.94	1.17	1.23
B9	1×10^{-6}	1×10^{-6}	0.0852	0.0884	0.0698	0.0784	0.0824	1.45	1.56	1.23	1.38	1.43
B10	9×10^{-7}	9×10^{-7}	0.0868	0.0887	0.0718	0.0797	0.0838	1.47	1.56	1.26	1.41	1.45
B11	8×10^{-7}	8×10^{-7}	0.0885	0.0891	0.0740	0.0813	0.0853	1.50	1.57	1.30	1.44	1.48

B12	7×10^{-7}	7×10^{-7}	0.0903	0.0894	0.0762	0.0827	0.0868	1.53	1.58	1.34	1.46	1.50
B13	6×10^{-7}	6×10^{-7}	0.0920	0.0903	0.0785	0.0848	0.0884	1.56	1.59	1.38	1.47	1.53
B14	5×10^{-7}	5×10^{-7}	0.0941	0.0902	0.0810	0.0861	0.0900	1.60	1.59	1.42	1.52	1.56
B15	4×10^{-7}	4×10^{-7}	0.0961	0.0905	0.0835	0.0879	0.0917	1.63	1.60	1.47	1.55	1.59
B16	3×10^{-7}	3×10^{-7}	0.0981	0.0910	0.0862	0.0898	0.0935	1.67	1.60	1.52	1.59	1.62
B17	2×10^{-7}	2×10^{-7}	0.1004	0.0913	0.0890	0.0917	0.0953	1.71	1.61	1.57	1.62	1.65
B18	1×10^{-7}	1×10^{-7}	0.1030	0.0917	0.0920	0.0937	0.0970	1.74	1.62	1.62	1.66	1.68
B19	9×10^{-8}	9×10^{-8}	0.1030	0.0917	0.0923	0.0939	0.0975	1.75	1.62	1.62	1.66	1.69
B20	8×10^{-8}	8×10^{-8}	0.1030	0.0918	0.0926	0.0942	0.0977	1.75	1.62	1.63	1.66	1.69
B21	7×10^{-8}	7×10^{-8}	0.1030	0.0918	0.0929	0.0944	0.0979	1.76	1.62	1.63	1.67	1.69
B22	6×10^{-8}	6×10^{-8}	0.1035	0.0918	0.0932	0.0946	0.0981	1.76	1.62	1.64	1.67	1.70
B23	5×10^{-8}	5×10^{-8}	0.1040	0.0919	0.0935	0.0948	0.0938	1.76	1.62	1.64	1.67	1.70
B24	4×10^{-8}	4×10^{-8}	0.1040	0.0919	0.0938	0.0950	0.0985	1.77	1.62	1.65	1.68	1.71
B25	3×10^{-8}	3×10^{-8}	0.1040	0.0919	0.0941	0.0952	0.0987	1.77	1.62	1.66	1.68	1.71
B26	2×10^{-8}	2×10^{-8}	0.1045	0.0920	0.0944	0.0954	0.0989	1.78	1.62	1.66	1.69	1.71
B27	1×10^{-8}	1×10^{-8}	0.1048	0.0920	0.0948	0.0957	0.0991	1.78	1.62	1.67	1.69	1.72

Table S3. Calculation Programs and Results (Q).

**Schemes**		**TP Concentration (mg/L)**					**TN Concentration (mg/L)**				
**Plan**	**Q（m^3/d）**	**SE**	**NE**	**SW**	**NW**	**Whole**	**SE**	**NE**	**SW**	**NW**	**Whole**
C1	350,000	0.0926	0.0902	0.0785	0.0844	0.0884	1.56	1.59	1.39	1.49	1.53
C2	319,680	0.0918	0.0900	0.0784	0.0851	0.0884	1.56	1.59	1.38	1.50	1.53
C3	298,080	0.0915	0.0903	0.0785	0.0846	0.0883	1.55	1.59	1.37	1.50	1.53
C4	267,840	0.0912	0.0903	0.0778	0.0845	0.0880	1.55	1.59	1.37	1.49	1.52
C5	246,240	0.0911	0.0900	0.0780	0.0843	0.0878	1.54	1.59	1.37	1.49	1.52
C6	216,000	0.0907	0.0900	0.0779	0.0840	0.0874	1.53	1.59	1.37	1.48	1.51
C7	185,760	0.0903	0.0900	0.0778	0.0840	0.0873	1.52	1.59	1.37	1.48	1.51
C8	155,520	0.0898	0.0899	0.0777	0.0836	0.0870	1.51	1.59	1.37	1.47	1.50
C9	125,280	0.0893	0.0898	0.0776	0.0833	0.0867	1.50	1.58	1.36	1.47	1.50
C10	103,680	0.0891	0.0898	0.0775	0.0831	0.0865	1.50	1.58	1.36	1.47	1.49
C11	82,080	0.0889	0.0897	0.0775	0.0827	0.0863	1.49	1.58	1.36	1.46	1.49

C12	60,480	0.0891	0.0896	0.0773	0.0826	0.0863	1.50	1.59	1.36	1.46	1.49
C13	43,200	0.0889	0.0896	0.0773	0.0820	0.0861	1.49	1.58	1.35	1.44	1.48
C14	25,920	0.0887	0.0896	0.0769	0.0813	0.0859	1.49	1.58	1.35	1.43	1.48
C15	12,960	0.0885	0.0896	0.0767	0.0808	0.0857	1.49	1.58	1.35	1.43	1.48
C16	0	0.0884	0.0896	0.0768	0.0800	0.0854	1.48	1.58	1.34	1.41	1.47

Table S4. Calculation Programs and Results (D).

Schemes			TP Concentration (mg/L)					TN Concentration (mg/L)				
Plan	D		SE	NE	SW	NW	Whole	SE	NE	SW	NW	Whole
	TP	TN										
D1	9	9	0.0918	0.0899	0.0784	0.0856	0.0884	1.56	1.58	1.37	1.51	1.53
D2	8	8	0.0918	0.0899	0.0784	0.0855	0.0884	1.56	1.58	1.37	1.51	1.53
D3	7	7	0.0919	0.0899	0.0784	0.0855	0.0884	1.57	1.58	1.37	1.51	1.53
D4	6	6	0.0919	0.0898	0.0784	0.0854	0.0884	1.57	1.58	1.37	1.51	1.53
D5	5	5	0.0920	0.0898	0.0783	0.0852	0.0884	1.57	1.58	1.38	1.51	1.53
D6	4	4	0.0920	0.0898	0.0784	0.0851	0.0884	1.57	1.58	1.38	1.50	1.53
D7	3	3	0.0921	0.0898	0.0784	0.0849	0.0884	1.57	1.58	1.38	1.50	1.53
D8	2	2	0.0921	0.0898	0.0785	0.0848	0.0884	1.57	1.58	1.38	1.50	1.53
D9	1.5	1.5	0.0921	0.0898	0.0785	0.0846	0.0884	1.57	1.58	1.38	1.49	1.53
D10	1	1	0.0920	0.0903	0.0785	0.0848	0.0884	1.56	1.59	1.38	1.47	1.53
D11	0.9	0.9	0.0921	0.0898	0.0785	0.0844	0.0883	1.56	1.58	1.38	1.49	1.53
D12	0.8	0.8	0.0921	0.0898	0.0786	0.0843	0.0883	1.56	1.58	1.38	1.49	1.53
D13	0.7	0.7	0.0921	0.0898	0.0785	0.0843	0.0883	1.56	1.58	1.38	1.49	1.53
D14	0.6	0.6	0.0921	0.0895	0.0785	0.0843	0.0883	1.56	1.58	1.38	1.49	1.53
D15	0.5	0.5	0.0921	0.0898	0.0785	0.0842	0.0883	1.56	1.58	1.38	1.49	1.53
D16	0.4	0.4	0.0921	0.0898	0.0785	0.0842	0.0883	1.56	1.58	1.38	1.49	1.53
D17	0.3	0.3	0.0921	0.0898	0.0785	0.0842	0.0883	1.56	1.58	1.38	1.49	1.53
D18	0.2	0.2	0.0921	0.0898	0.0785	0.0841	0.0883	1.56	1.58	1.38	1.49	1.53
D19	0.1	0.1	0.0921	0.0898	0.0785	0.0841	0.0882	1.56	1.58	1.38	1.49	1.53
